# Clinical application of radiotherapy in patients with oligometastatic ovarian cancer: a sharp tool to prolong the interval of systemic treatment

**DOI:** 10.1007/s12672-022-00540-y

**Published:** 2022-08-25

**Authors:** Jing Shen, Yinjie Tao, Lei He, Hui Guan, Hongnan Zhen, Zhikai Liu, Fuquan Zhang

**Affiliations:** Department of Radiation Oncology, Peking Union Medical College Hospital. Chinese Academy of Medical Sciences & Peking Union Medical College, NO.1 Shuaifuyuan Wangfujing, Dongcheng District, Beijing, 100730 People’s Republic of China

**Keywords:** Ovarian cancer, Oligometastasis, Intensity modulated radiation therapy, Delaying systemic treatment time

## Abstract

**Background:**

With the advances of radiation technology, treatment of oligometastatic disease, with limited metastatic burden, have more chances to achieve long-term local control. Here we aim to evaluate the efficacy and safety of radiotherapy (RT) in oligometastatic ovarian cancer patients.

**Methods:**

A retrospective analysis collecting 142 patients (189 lesions) with oligometastatic ovarian cancer were included in the study. All pateints received radiotherapy and the curative effect and response rate were evaluated by diagnostic imaging after 1–3 months of radiotherapy with RECIST. Endpoints were the rate of complete response (CR), chemotherapy-free interval (CFI), local control (LC) rate and overall survival (OS) rate. Toxicity was evaluated by the Radiation Therapy Oncology Group (RTOG). Logistic and Cox regression were used for the uni- and multivariate analysis of factors influencing survival outcomes.

**Results:**

From 2013.1.1 to 2020.12.30, a total of 142 ovarian cancer patients (189 oligometastasis lesions) were included in the analysis. Prescribed doses to an average GTV of 3.10 cm were 1.8–8 Gy/fraction, median BED (28–115, a/b = 10 Gy), 5–28 fractions. For 179 evaluable lesions, the cases of CR, partial response (PR), stable disease (SD) and progressive disease (PD) after radiotherapy were 22,39,38 and 80 respectively. The disease control rate (DCR): CR + PR + SD was 55.31%, and the objective response rate (ORR): CR + PR was 34.08%. No patient developed grade 3 or higher side effect. The median CFI was 14 months (1–99 months), and the LC rate was 69.7%, 54.3% and 40.9% in 1 year, 2 years and 5 years respectively. GTV < 3 cm before treatment, platinum sensitivity, time from the last treatment ≥ 6 months, single lesion and BED(a/b = 10 Gy) ≥ 60 are the factors of good LC (p < 0.05). The total OS of 1 year, 2 years and 5 years were 67.1%, 52.6% and 30.3%, respectively. Single lesion (HR 0.598, 95%CI 0.405–0.884), DCR (HR 0.640, 95% CI 0.448–0.918) and ORR(HR 0.466, 95% CI 0.308–0.707) were the significant factors influencing 5-year OS.

**Conclusion:**

For patients with oligometastatic ovarian cancer, radiotherapy has high LC, long chemotherapy-free interval, and survival benefits. Subgroup analysis shows that patients with single lesion and good local treatment results have higher overall survival rate, suggesting that active treatment is also beneficial for oligometastatic ovarian cancer patients.

## Introduction

Ovarian cancer is the third most prevalent malignant tumor of the female reproductive system [1]. The five-year mortality rate of ovarian cancer exceeds 50%, according to the 2018 GLOBOSCAN study of the International Agency for Research on Cancer (IARC) [2]. The global ovarian cancer burden is expected to increase by 47% between 2018 and 2040 (295,414 to 434,184 women) [3]. Because of the oblique signs of ovarian cancer and the lack of efficient screening, roughly 75% of women with the disease are locally advanced at the time of diagnosis (stage III–IV). Even after receiving routine surgery and adjuvant chemotherapy, more than 70% to 80% of patients with locally advanced ovarian cancer will recur or metastasize [4].

Oligometastasis refers to a condition with 1–5 metastatic lesions, lacking the potential to develop widespread the whole body. The term means a transitional stage between localized and metastatic disease. Previous studies have shown the promising results of RT in the clinical setting of oligometastatic malignancies, such as nonsmall-cell lung cancer (NSCLC) and prostate cancer [5, 6]. Multiple reports has proven the definitive role of radiotherapy in the treatment and control of oligometastatic disease [7–9].

The widely used intensity modulated radiation therapy (IMRT) brought radiation to a new level. By using a three-dimensional imaging technology and uniformly focusing the high dosage into the target area, the irradiation dose can be increased while the dose to normal tissues and organs is reduced as much as feasible. Higher-dose radiation in patients with oligometastatic ovarian cancer thus becomes a reality, attracting much attention [10, 11]. Modern RT modality, such as hypo-fractionated radiation also provided more chances to achieve a high local control rate, extend the chemotherapy-free interval (CFI), and improve survival outcomes [4, 7–9, 12]. Multiple prospective trials are ongoing investigating the efficacy and safety of RT in oligometastatic ovarian cancer [13].

Here, we mainly investigated the treatment role of RT in oligometastatic ovarian cancer and reviewed the past 10-year experience in our institution. The treatment, local control rate, chemotherapy-free interval, survival benefit, and toxic and side effects were reported and analyzed.

## Materials and methods

### Patients

We retrospectively collected ovarian cancer patients treated in the radiotherapy department of Peking Union Medical College Hospital from January 2013 to December 2020. This study was reviewed and approved by the ethics committee of Peking Union Medical College Hospital in accordance with the Declaration of Helsinki and relevant policies in China.

A total of 142 patients (189 lesions) were included, provided that they respected the following criteria:

- Pathologically proven primary ovarian cancer, already underwent at least one standard cytoreductive surgery and received at least one full course of systemic therapy (including chemotherapy, immunotherapy, and targeted therapy);
- Less than 5 newly discovered metastatic lesions based on clinical examination, laboratory examination and imaging examination (including CT, MRI, and PET-CT);
- Inappropriate for recytoreductive surgery or systemic therapy due to contra-indications or patient refusal;
- General condition not too poor for radiotherapy.

All patients underwent a standard restaging process before submitting to radiotherapy, including history collection, physical examination, laboratory sample test, and total body CT with contrast and FDG-PET. The largest diameter of measurable lesions is measured to standardize the location of metastases. Patients with any site of metastases were considered acceptable for inclusion. All of the patients signed an informed consent before the treatment.

### Radiotherapy

Radiotherapy was located by CT simulation (16-slice Philips Bril, Liance CT Big Bore, Deventer, Netherlands). All patients were given appropriate fixation devices according to the target site. Oral and intravenous contrast agents were taken if necessary.

Gross tumor volume (GTV) and clinical target volume (CTV) are outlined and delineated on axial CT slices. GTV includes metastatic tumor lesion, which is determined by CT, MRI and PET-CT. CTV covers GTV and surrounding high-risk areas. The planning gross tumor volume (PGTV) was generated from GTV by adding a fixed distance of 5 mm margin in all directions, and the planning target volume (PTV) was generated from CTV expanding a flexible margin of 3–8 mm according to PGTV and the surrounding OARs. Patients were given PTV with a dose of 1.8 to 8 Gy, 5–28 fractions and a prescription dose of 24–50 Gy. The synchronous dosage of PGTV was 40–60 Gy. The plan objective was to cover at least 95% of prescription dose to 95% of PTV and PGTV. The radiotherapy plan was generated on Eclipse or tomotherapy planning system. Dose constraints of organ at risk (OARs) were strictly abided by International Commission Radiological Units (ICRU)’s hazardous OAR limit. Priority was given to the OARs if there was overlap between target dose and normal tissue.

In order to ensure the exact accuracy of positioning during the treatment period, the images of megavoltage computed tomography (MVCT) for patients receiving IMRT with spiral tomography (tomotherapy) were collected on a daily basis and registered online. For patients who received fixed-filed intensity modulated radiotherapy (FF-IMRT) or volumetric-modulated arc therapy (VMAT), image guidance was performed by matching cone beam CT (CBCT) images online every week within an error of 3 mm. Techniques for respiratory movement control were not applied in this study due to the heterogeneity of metastatic sites in these patients.

### Follow-up and efficacy evaluation methods

Patient baseline and clinicopathological characteristics were collected, including gender, age, blood tumor markers before treatment, operation stage, whether initial treatment, prior treatment, platinum sensitivity (a response to chemotherapy ≥ 6 months), recurrence times, blood tumor markers at the time of recurrence, and treatment methods after recurrence, etc.

Through retrieving and consulting medical records and telephone follow-up, patients were followed up once every 3 months within 2 years after treatment and once every 6 months for 2–5 years. The follow-up period was from the end date of radiotherapy treatment to the last follow-up date or death time, and the follow-up deadline was December 30th, 2021. The tumor response was evaluated by diagnostic imaging (CT, MRI and PET-CT) according to the standard curative effect of solid tumor treatment (RECIST) version 1.1. Long-term follow-up contents include survival status, overall survival time, progression free survival time, time without local recurrence, and chemotherapy-free interval. Overall survival (OS) is defined as the time from completion of radiotherapy to the last follow-up date or the time of death. Progression free survival (PFS) is defined as the time from completion of radiotherapy to the first occurrence of disease progression. Local recurrence free survival (LRFS) is defined as the time from the completion of radiotherapy to the occurrence of local recurrence in the irradiation of radiotherapy. Chemotherapy-free interval (CFI) is calculated from the date of completion of radiotherapy to the first date of administration of the next chemotherapy course or the last follow-up.

The toxic and side effects were evaluated by Common Terminology Criteria for Adversity Events (CTCAE) version 4.0.

### Statistical methods

Chi-square test was used to classify variables. The normality of continuous variables was tested by Kolmogorov–Smirnov method. Student-T test was used to evaluate normally distributed variables and Mann–Whitney U test was used for non-normally distributed variables. The end-points of the study were LC, PFS, OS and CFI. The incidence of OS, PFS, LRFS were estimated by Kaplan–Meier method and univariate logarithmic rank test was used to evaluate the significance of prognostic factors to survival rate. Cox proportional hazard regression method was used to analyze the covariates selected from univariate analysis. P < 0.05 was considered to be significant.

The short-term curative effect was analyzed ‘per lesion’ and the long-term survival outcomes were analyzed ‘per patient’.

SPSS version 25.0 was used for statistical analysis.

## Results

### Patients’ characteristics

**Fig. 1:**
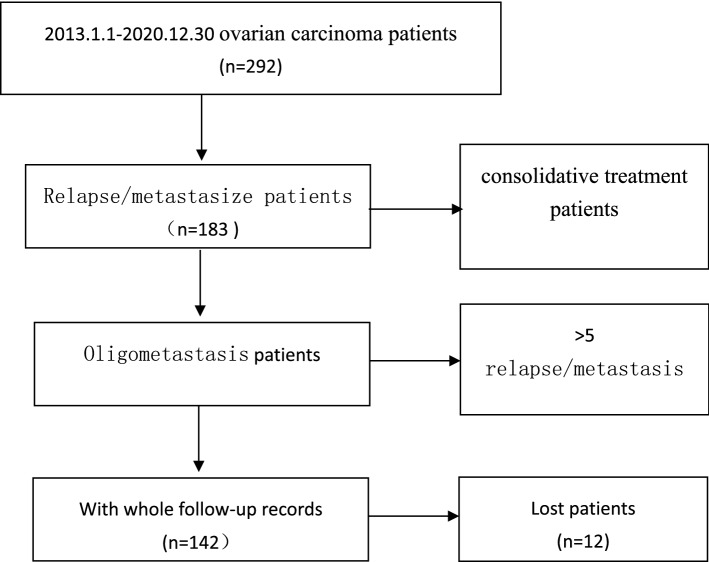
Flowchart of patients selection

A total of 142 patients with 189 metastatic lesions were enrolled according to the inclusion criteria (Fig. 1). The patients’ age ranged from 21 to 79 years, with an average age of 56 years. There were 132 cases of epithelial carcinoma (101 serous, 12 endometrial, 13 clear cell and 6 other epithelial carcinoma), 6 cases of sex cord stromal and 4 cases of germ cell tumor. The median time to the last treatment was 12.77 months. Overall, 67 cases were presented with single lesion and 75 with 2 or more metastatic lesions. The majority of the patients (n = 129, 90.8%) developed metastasis outside visceral organs, such as lymphatic drainage area. All patients already submitted to upfront surgery and chemotherapy. Patient baseline and clinicopathological information were demonstrated in Table 1.

**Table 1:** Baseline and clinical characteristics of the patients (n = 142)

Characteristics	Number (n = 1)	%
Age(years)		
< 60	91	64.1
≥ 60	51	35.9
KPS score		
70–80	20	14.1
90–100	122	85.9
Histology		
Epithelial carcinoma	132	93.0
Serous	101	71.17
Endometrial	12	8.45
Clear cell carcinoma	13	9.15
Others	6	4.23
Interstitial tumors of the sex cords	6	4.2
Germ cell tumors	4	2.8
FIGO stage		
I	12	8.5
II	13	9.2
III	102	71.7
IV	15	10.6
Recurrence/metastasis times		
< 2	84	59.2
≥ 2	58	40.8
Time to the last treatment		
< 6 months	35	24.6
≥ 6 months	107	75.4
Platinum sensitive station		
PFI ≥ 6 months	20	14.1
PFI < 6 months	122	85.9
Recurrence/metastasis lesion		
Single lesion	67	47.2
2 lesions	51	35.9
3–5 lesions	24	16.9
Recurrence/metastasis lesion		
Visceral organ	13	9.2
Non visceral organ	129	90.8
Prior treatment		
Operation	142	100
Chemotherapy	142	100
Immune therapy	20	14.08
Target therapy	24	16.90

*FIGO* international federation of gynecologists and obstetrics, *PFI* platinum-free interval, *RT* radiation therapy

The characteristics of 189 lesions and RT treatment towards local lesions were also demonstrated in Table 2. Retroperitoneal lymphatic drainage area and neck lymph nodes were two most common metastatic sites. The average doses prescribed were 1.8–8 Gy/fraction (median BED 68, a/b = 10 Gy), either hypofractionated or regular fractionated according to the metastatic site. The average diameter of GTV was 3.10 cm. All patients completed the prescribed treatment.

**Table 2:** Characteristics of lesions

Characteristics	n	%
Radiation lesions		
Visceral organ	21	11.1
Non visceral organ	168	88.9
The number of recurrent/metastatic lesions		
< 2	67	35.4
2–3	82	43.4
4–5	40	21.2
Distribution of lesions		
Intracranial	13	6.9
Neck	38	20.1
Mediastinum	17	9.0
Bone	9	4.8
Liver	3	1.6
Abdominal cavity	12	6.3
Pelvic cavity	17	9.0
Retroperitoneal lymphatic drainage area	48	25.4
Vaginal stump	27	14.3
Prior treatment time		
< 6 months	44	23.3
≥ 6 months	145	76.7
GTV diameter(cm)		
< 3	114	60.32
≥ 3	65	34.39
Unknown	10	5.29
BED(a/b = 10 Gy)		
< 60	36	20.11
≥ 60	143	79.89
BED		
Median	68.04	
Range	28–115	
GTV		
Median	3.10	
Range	1–12.30	

*BED* biologically effective dose, *GTV* gross tumor volume

### Treatment results

#### Short-term efficacy evaluation

**Fig. 2:**
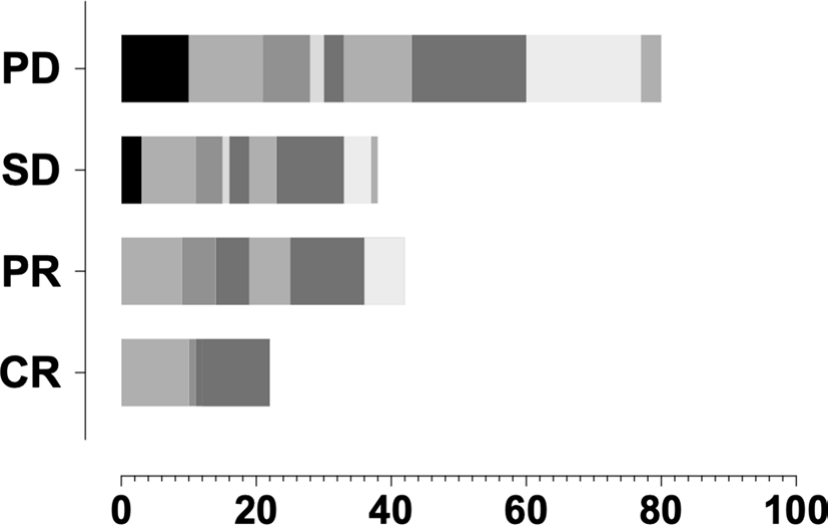
Short-term curative effect

The short-term efficacy was analyzed ‘per lesion’. According to the RECIST standard, the curative effect could be measured in 179 lesions. The evaluation began 1–3 months after RT based on diagnostic imaging and the average interval after RT was 50 days. Among them, 22 lesions were CR, 39 PR, 38 SD and 80 PD. The DCR (CR + PR + SD) was 55.3% and the ORR (CR + PR) was 34.1%. Lymph nodes in neck and retroperitoneal region had the most cases of CR. See Table 3 and Fig. 2 for details.

**Table 3:** Short-term efficacy evaluation

Lesions	CR	PR	SD	PD	Total
Intracranial	0	0	3	10	13
Neck	10	9	8	11	38
Mediastinum	1	5	4	7	17
Liver	0	0	1	2	3
Abdominal cavity	1	5	3	3	12
Pelvic cavity	0	3	4	10	17
Retroperitoneal lymphatic drainage area	10	11	10	17	48
Vaginal stump	0	6	4	17	27
Others	0	0	1	3	4
Total	22	39	38	80	179

#### Long-term curative effect analysis

**Fig. 3:**
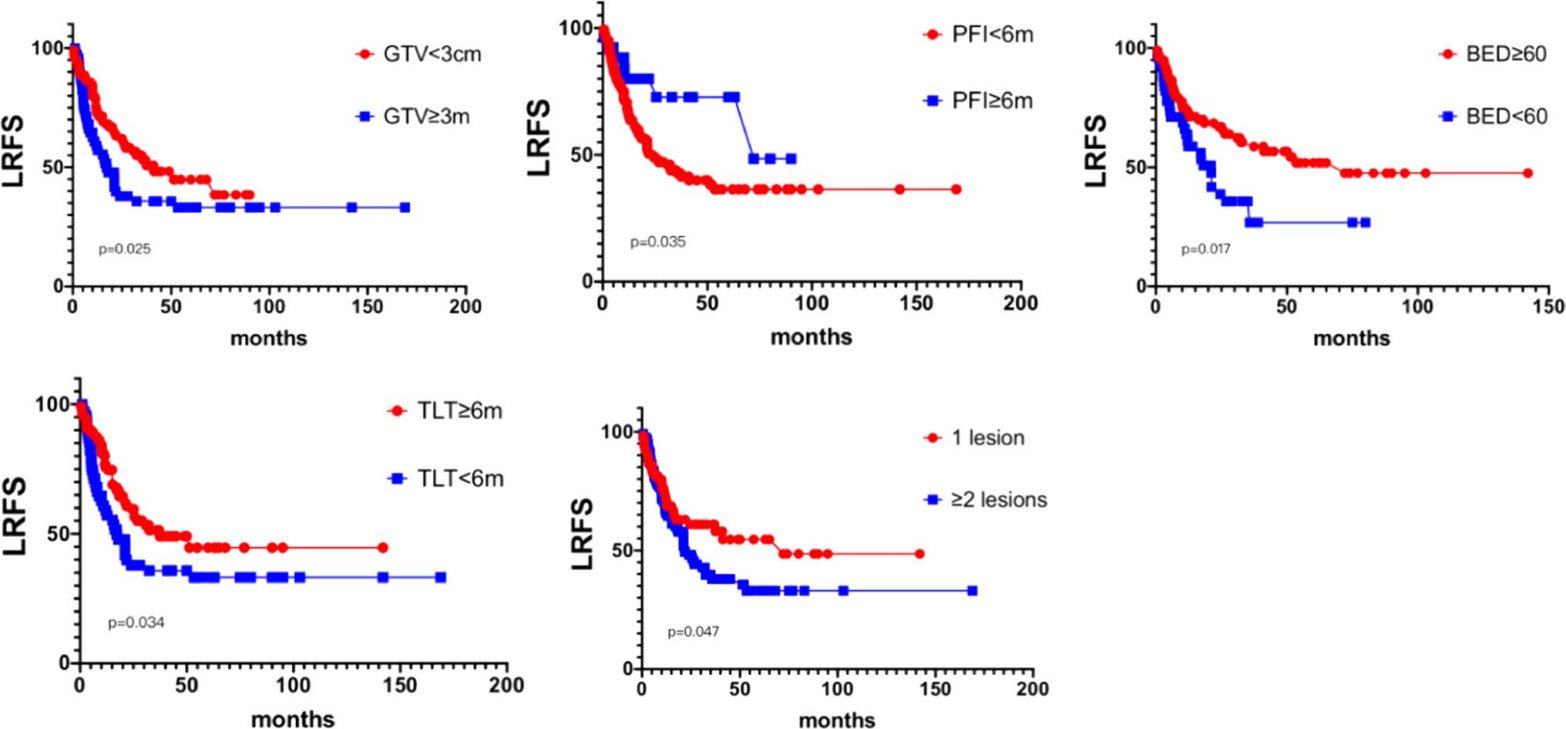
LRFS and subgroup analysis. LRFS: Local Recurrence Free Survial, GTV, PFI, TLT,BED

The 1-, 2- and 5-year local control rate were 69.7%, 54.3% and 40.9% respectively. GTV < 3 cm before treatment (63.60% v.s. 49.45%, p = 0.025), platinum sensitivity (71.32% v.s. 64.23%, p = 0.035), time to last treatment ≥ 6 months (90.76% vs 66.94%, p = 0.034), single lesion (79.23% v.s. 66.55%, p = 0.047), and BED (a/b = 10 Gy) ≥ 60 (81.40% v.s. 28.96%, p = 0.07) were good factors of LRFS. See Fig. 3 below for details.

**Fig. 4:**
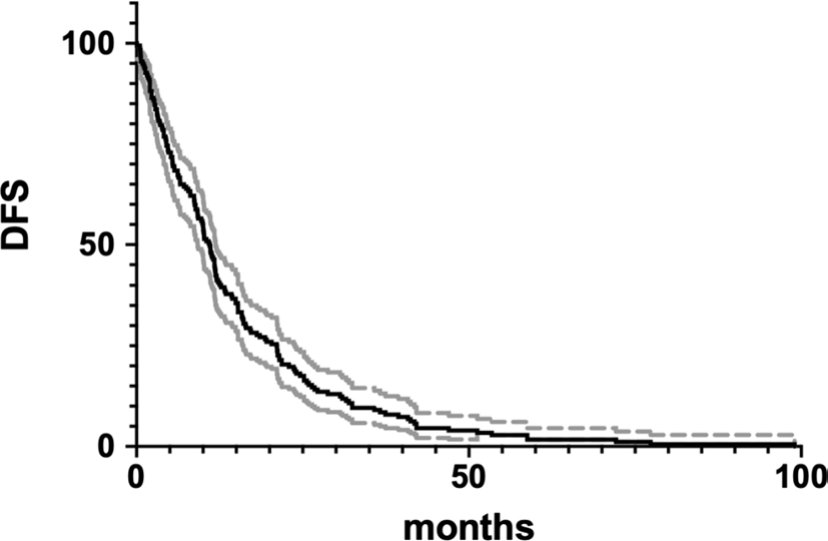
Kaplan–Meier curve for progression free survival (PFS) of the 189 treated lesions over time (solid line). The dashed lines indicate 95% confidence intervals

The long-term survival outcomes were analyzed ‘per patient’. The median progression time of 142 patients after RT was 11 months (1–99 months), as shown in Fig. 4. The 1-, 2- and 5-year PFS rates were 40.5%, 17.8% and 1.6% respectively. The median delay of systemic treatment time was 14 months (1–99 months).

**Fig. 5:**
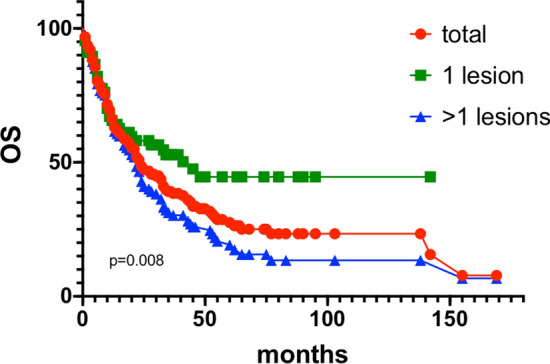
OS and subgroup analysis (number of lesions

For all 142 patients, the 1-, 2-, and 5-year OS rate were 67.1%, 52.6% and 30.3% respectively. For patients who had only one lesion, the 1-, 2-, and 5-year overall survival rate were 67.2%, 58.1%, and 44.6% respectively. The OS rate dropped significantly to 66.7%, 42.9%, and 20.6% respectively for those who had 2 or more metastatic lesions, p = 0.008. Details were depicted in Fig. 5.

**Fig. 6:**
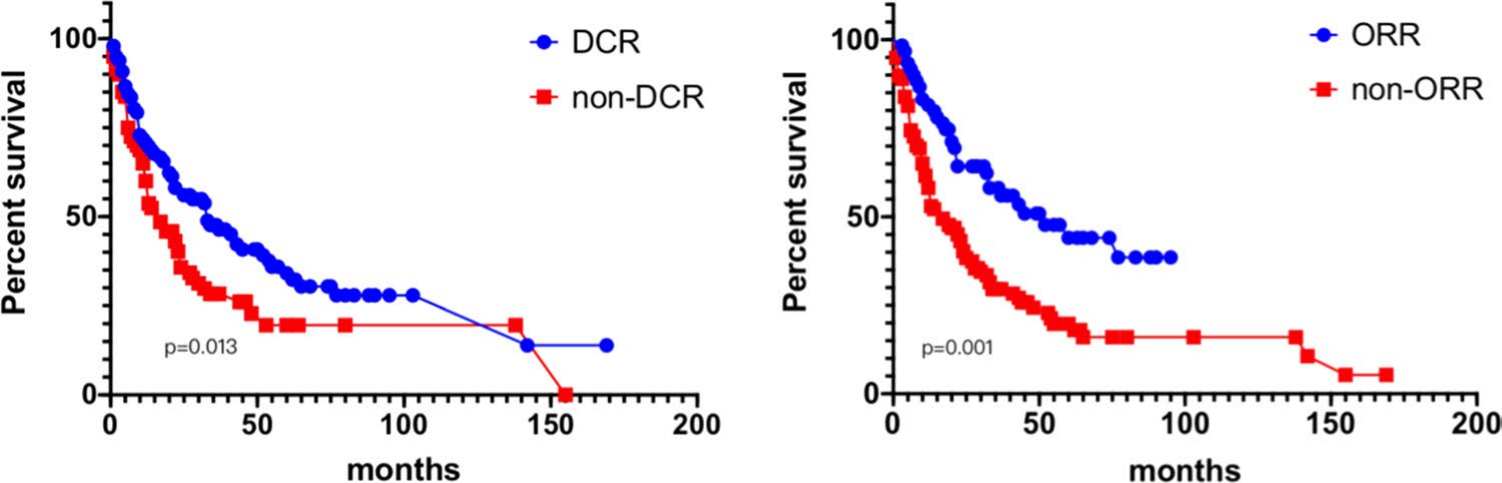
Overall OS and subgroup analysis (DCR and ORR

The OS for those patients who achieved DCR was higher, with 1-year, 2-year and 5-year OS of 71.1% (v.s. 60.0%), 58.4% (v.s. 40.2%), and 36.1% (v.s. 19.6%), respectively, p = 0.013. Similarly, The OS for those patients who achieved ORR were also higher, with 1-year, 2-year and 5-year OS of 83.2% (v.s. 61.6%), 64.5% (v.s. 40.3%), and 47.8% (v.s. 19.8%), respectively, p = 0.001 (Fig. 6).

#### Toxic and side effects

The radiotherapy was well tolerated, with 98 patients having no acute or chronic toxic and side effects. No grade 3 or above toxic and side effects reported. Twenty-five patients had acute reactions, including: 19 cases of acute grade 1–2 gastrointestinal reactions, and 16 cases of grade 2 hematological toxicity. Chronic reactions were observed in 20 patients: 16 cases of chronic 1–2 grade gastrointestinal reactions and 4 cases of grade 1–2 myelosuppression.

#### Univariate and multivariate analysis

Univariate and multivariate analysis showed that the number of metastatic lesions (HR 0.598, 95% CI 0.405–0.884, p = 0.010), DCR (HR 0.640, 95% CI 0.448–0.918, p = 0.015), and ORR (HR 0.466, 95% CI 0.308–0.707, p = 0.001) were the related significant factors of the overall survival. The detailed information were provided in Table 4.

**Table 4:** Univariate and multivariate analysis of factors influencing 5-year OS

Characteristics	Univariate analysis	Multivariate analysis		
		HR	95% CI	p value
Histology	0.117			
Epithelial carcinoma	33.1			
interstitial tumors of the sex cords	30.0			
Germ cell tumors	0			
FIGO stage	0.247			
I	52.5			
II	52.44			
III	58.94			
Recurrence/metastasis times	0.227			
< 2	59.76			
≥ 2	48.22			
Time to the last treatment	0.309			
< 6 months	44.42			
≥ 6 months	57.62			
Platinum sensitive station	0.338			
PFI ≥ 6 m	55.16			
PFI < 6 m	32.23			
Recurrence/metastasis lesion	0.008*	0.598	0.405–0.884	0.010*
Single	72.29			
≥ 2	41.79			
Recurrence/metastasis lesion	0.074			
Visceral organ	24.94			
Non visceral organ	56.76			
GTV diameter	0.208			
< 3 cm	51.50			
≥ 3 cm	48.62			
BED(a/b = 10 Gy)	0.165			
< 60	28.60			
≥ 60	55.51			
DCR	0.013*	0.640	0.448–0.918	0.015*
Yes	62.01			
No	43.87			
ORR	0.001*	0.466	0.308–0.707	0.001*
Yes	54.42			
No	41.06			

## Discussion

Oligometastases, referring to the condition when metastatic lesions are limited (usually no more than 5), are hot-spot for researchers in many types of cancer. The concept was isolated from high-volume metastases because these patients appeared to have better outcomes and were clinically worthy of higher grades of intervention [14]. For these patients, recent years have witnessed the transition of the treatment modality, from trying further lines of chemotherapy, to more radical local control methods, such as recytoreductive surgery or stereotactic body radiation [15–17]. The former old-fashioned treatment option had limited benefits for ovarian cancer patient: even for patients sensitive to platinum, the recurrence rate of second-line chemotherapy remained high, at 50–60% [18, 19].

Different treatment plans are applied to patients with oligometastatic ovarian cancer based on the location and number of lesions, chemotherapy sensitivity or whether cytoreductive surgery is to be conducted again. Ovarian malignant tumors were relatively sensitive to radiotherapy, especially asexual tumor cell, epithelial tumor cell, and granular cell carcinoma [20]. Despite the limited application of radiotherapy in ovarian cancer patients in current clinical guidelines [11], radiotherapy was still an acceptable alternative for local control. Patients with isolated metastasis or localized recurrence could achieve a good local control rate by radiotherapy [21, 22]. Moreover, radiation was less invasiveness and more tolerable in patients unsuitable for surgery.

The literature reports of radiation for oligometastatic ovarian cancer in recent years were still limited, as summarized in Table 5 [7, 9, 15, 23–28]. All of the patients received three-dimensional or IMRT. For normal fractionated radiotherapy, the radiation dose was 50.4–60.6 Gy, and the CR rate ranged from 64.3 to 85.0% for local lesions. Stereotactic body radiotherapy (SBRT) was a representative cutting edge of modern RT, which could deliver high doses to small volumes of metastatic sites in few fractions and be employed as a part of curative-intent treatment strategies [29, 30]. Three important articles regarding hypo-fractionated SBRT were listed. The CR rate ranged from 59.1 to 65.2% per lesion and 2-year LC was acceptable (81.9 to 92.9%). No 3–4 grade side effects were found.

**Table 5:** Literature reports review

Year	Author	Number	Lesions	Treatment	Short-time effect	Side effect	Long time effect
2001[19]	Firat et al	28	Vaginal stump or pelvic cavity	21例EBRT 50.4 Gy	57%CR	–	50%patients clinical symptom relief
2002[20]	Fujiwara K et al	44	Abdominal cavity,pelvic cavity,retroperitoneal lymphatic drainage area,vaginal stump	EBRT 52.3 ± 8.3 Gy	–	No grade 3 side effects	Survival benefit of asymptomatic patients and patients with lymph node metastasis after radiotherapy
2005[21]	Albuquerque et al	20	Abdominal cavity	EBRT 50.4 Gy	85%CR	3 patients with 3-4GI effect	5 year LRFS 66% OS34% DFS 34%
2011[22]	Shin-Wha Lee et al	38	Abdominal cavity,pelvic cavity,retroperitoneal lymphatic drainage area	EBRT 50.4 Gy	65%CR	No grade 3 side effects	PFS 7.2 months
2013[23]	Yahara K et al	27	Abdominal cavity,pelvic cavity,retroperitoneal lymphatic drainage area	EBRT 60 Gy	–	No grade 3 side effects	2 year OS 53% PFS 39% LRFS 96%
2013[24]	Brown et al	102	Abdominal cavity,pelvic cavity,retroperitoneal lymphatic drainage area	EBRT 59.2 Gy(45–68.2)	–	No grade 3 side effects	5 year LRFS 71% OS40% DFS 24%
2014[25]	Saito M et al	61	Abdominal cavity,pelvic cavity,retroperitoneal lymphatic drainage area	EBRT 60 Gy(15.6–72)	–	No grade 3 side effects	Rest period and radiotherapy response rate were related to survival
2016[26]	Albuquerque K et al	27	Abdominal cavity,pelvic cavity,retroperitoneal lymphatic drainage area	EBRT 50.4 Gy	70%CR	No grade 3 side effects	10 year LRFS 60% OS19% DFS 20%
2017[27]	Choi N et al	47	Retroperitoneal lymphatic drainage area,intracranial,bone,pelvic cavity	BED 50.7 Gy(28.0–79.2)	66.7%CR	No grade 3 side effects	PFS 16.2 months

Our results of outcomes were relatively lower compared with the previous studies. The limits largely contributed to the difference of treatment intention, and heterogeneity over the long time of advancing radiation technique employed, and imaging for assessment of clinical response. As a single-institution retrospective study, selection bias was inevitable. Inclusion of large (lesions > 5 cm) lesions and all metastatic sites also played a role. In the study reported by Iftode et al., only extracranial lesions were included, for example [7]. Sill, we had some interesting findings. Lymph node lesions in neck and retroperitoneal region showed a higher responsiveness to radiation compared with visceral or parenchymal lesions, which was in line with the previous studies [8, 16]. Intracranial lesions and metastatic sites on liver, vaginal stump were possibly more resistant to radiation therapy, as CR were quite low under these conditions. The target doses might be sacrificed to protect OARs. Detailed comparative studies and further investigations into the biological mechanisms were needed.

As far as the factors influencing LC rate, we found multiple factors related to good LC. Platinum sensitivity was paralleled with radiation sensitivity in the findings, which was also observed in the study of involved-field radiation therapy for recurrent ovarian cancer [22, 26]. BED (α/β = 10) above 60 Gy was associated to a higher response rate, similar to the previous reports regarding metastatic solid tumors [31, 32]. Tumors with GTV > 3 cm and 2 or more lesions were less likely to achieve local control, representing a higher volume and heavier load of metastatic carcinoma. For these disease, restaging before RT by a clear whole-body imaging is necessary to avoid costly local therapy for such condition which tends to be more widespread.

Regarding the toxic effect, there were no grade 3–4 side effects observed in our research and the literature we reviewed. Local RT was safe for those who could not tolerate or benefit from further series of chemotherapy or aggressive surgery.

In the current study, a total of 10 patients (10 lesions) were treated with SBRT, including 3 cases in liver, 1 case in lung, and 3 cases in other parts. The single fraction dose was 5–8 Gy, and the number of fractions was 6–10 times. Clinical efficacy reported 3 cases in PR, 5 cases in SD and 2 cases in PD. The median OS were 24 months. Compared with the normal fractionated RT, SBRT owned the advantages of stimulating activity in chemo-resistant disease and potential to mount immune response after cell killing by radiation [33–36]. SBRT has been an important treatment option to improve patients’ outcomes in several metastatic solid tumors, and the recent studies mostly focused on oligometastatic NSCLC and prostate cancer [37, 38]. More well-designed comparative studies are needed to guide the application of SBRT in oligometastatic ovarian cancer, to determine the optimal dose and fractionation for different metastatic sites, size and number of lesions we encountered an opportunity where RT served as a palliative tool to a curative tool to benefit patients (in scopes of prolonging interval of chemotherapy, overall survival time, progression-free survival time, etc.).

This study is a retrospective, single-center experience. Though the treatment is heterogeneous, this study is the largest published clinical retrospective study of palliative radiotherapy in ovarian oligometastasis up to now.

In conclusion, RT could be considered as a therapeutic option with mild toxicity for local control. Accurate choice of patients before RT and a better defined radiation scheme are needed for further research. Moreover, the combination of RT with radiotherapy sensitizer, targeted drugs and immunotherapy is also the focus of the related research.

## Data Availability

The data were available on reasonable request from the corresponding author.

## Abbreviations

RT: Radiotherapy
CR: Complete response
CFI: Chemotherapy-free interval
LC: Local control
OS: Overall survival
PR: Partial response
SD: Stable disease
PD: Progressive disease
DCR: Disease control rate
ORR: Objective response rate
NSCLC: Nonsmall-cell lung cancer
IMRT: Intensity modulated radiotherapy
GTV: Gross tumor volume
CTV: Clinical target volume
PTV: Planning target volume
PGTV: Planning gross tumor volume
OAR: Organ at risk
MVCT: Megavoltage computed tomography
FF-IMRT: Fixed-filed intensity modulated radiotherapy
VMAT: Volumetric-modulated arc therapy
CBCT: Cone beam CT
PFS: Progression free survival
LRFS: Local recurrence free survival
SBRT: Stereotactic body radiotherapy

## Code availability

Source code is available upon request from the corresponding author.
